# Growth-defense tradeoff in rice: The role of gibberellic acid catabolism

**DOI:** 10.1093/plcell/koad206

**Published:** 2023-07-25

**Authors:** Lucas Frungillo

**Affiliations:** Assistant Features Editor, The Plant Cell, American Society of Plant Biologists, Rockville, MD, USA; Institute of Molecular Plant Sciences, School of Biological Sciences, University of Edinburgh, Edinburgh, UK

Upon herbivore attack, phytohormonal crosstalk mediates metabolic reprogramming to prioritize defense responses over growth (Erb and Reymond 2019). However, how cellular resource allocation is wired into an effective defense response in plants is still largely unknown. In this issue of *The Plant Cell*, **Gaochen Jin and colleagues** (Jin et al. 2023) investigate mechanisms underlying the growth-defense tradeoff during herbivore attack. This work reveals novel players controlling plant growth and defense and shows that resistance against herbivores can be achieved without growth penalties by engineering complex hormonal networks.

**Figure 1.**
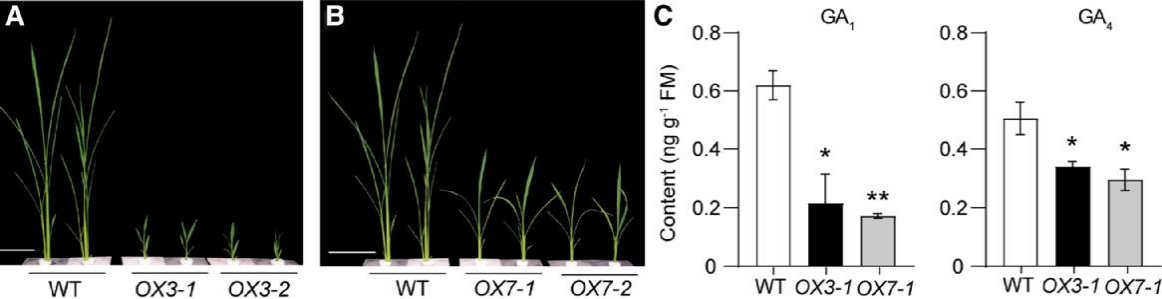
GA2ox3 and GA2ox7 promote growth restrictions on rice plants by regulating levels of bioactive gibberellic acid. **A, B)** Growth phenotype of 2 independent GA2ox3 (*OX3-1*, *OX3-2*) and GA2ox7 (*OX7-1*, *OX7-2*) overexpressor lines along with wild-type (WT) plants. Four-week-old seedlings were used. Scale bar = 10 cm. **C)** Levels of bioactive GA in GA2ox overexpressors and WT plants. FM, fresh mass. Asterisks indicate significant differences between overexpressors and WT plants (*P < 0.05; **P < 0.01; Student's *t* test). Adapted from Jin et al. (2023), Figure 2.

The brown planthopper (BPH), *Nilaparavata lugens*, is a major threat to rice (*Oryza sativa*). By sucking the sap from the phloem, BPH deprives rice plants of nutrients and causes serious yield losses (Xu et al. 2021). To fend off attackers, plants must detect salivary proteins secreted by BPH during feeding. Recent evidence indicates that recognition of herbivore-associated molecular patterns triggers changes in hormonal signaling pathways in plants (Gao et al. 2022). Because the phytohormone gibberellic acid (GA) regulates plant growth and development, the authors sought to investigate the impact of BPH feeding on GA levels in rice. Hormonal profiling by liquid chromatography–mass spectrometry revealed lower levels of GA bioactive forms in BPH-attacked rice compared with control plants. Accordingly, overexpression of the GA catabolic genes *GA2ox3* and *GA2ox7* resulted in lower levels of bioactive GA and reduced growth compared with wild-type plants (see Fig. 1), suggesting that herbivore attack triggers the catabolism of bioactive GAs in rice.

Next, the authors asked whether GA2ox3 and GA2ox7 mediate growth penalties in plants attacked by BPH. Interestingly, while *ga2ox3 ga2ox7* double mutants displayed reduced growth penalties in response to BPH elicitation, BPH performance assays revealed that *ga2ox3 ga2ox7* mutation did not affect BPH reproduction compared with wild-type plants. These exciting findings indicate that *GA2ox3* and *GA2ox7* mediate growth inhibition, but not defense, in response to BPH attack.

So how do plants coordinate resource allocation during defense? Rice defense against BPH is largely mediated by the phytohormone jasmonic acid (JA). Intriguingly, both JA and GA signaling recruit DELLA repressor proteins to tune activity of growth-promoting transcription factors (TFs), suggesting that crosstalk between JA-GA underpins tradeoffs during BPH attack. Therefore, the authors hypothesized that BPH-elicited GA catabolism is located downstream of JA signaling. Pharmacological treatments with bioactive JA upregulated *GA2ox3* and *GA2ox7* expression, as well as increased levels of their enzymatic activity. Additionally, overexpression of the JA master regulator, the TF MYC2, resulted in reduced levels of bioactive GA and reduced growth compared with wild-type plants. MYC2 belongs to the bHLH family of TFs that bind to G-box and G-box-like DNA motifs (Chini et al. 2007). Co-transfection and chromatin immunoprecipitation sequencing assays revealed that MYC2 binds to G-box and G-box–like motifs to directly regulate *GA2ox3* and *GA2ox7* transcription.

Collectively, Jin et al. (2023) provide compelling evidence that the MYC2-GA2ox module orchestrates JA-GA hormonal crosstalk in rice during defense responses against BPH attack. The mechanistic insights presented in this study open exciting new directions for the investigation of hormonal crosstalk in plants and provide a framework for improving resistance to pests without yield penalties in rice.
